# Independent association of resting energy expenditure with blood pressure: confirmation in populations of the African diaspora

**DOI:** 10.1186/s12872-017-0737-5

**Published:** 2018-01-10

**Authors:** Chloe Creber, Richard S. Cooper, Jacob Plange-Rhule, Pascal Bovet, Estelle V. Lambert, Terrence E. Forrester, Dale Schoeller, Walter Riesen, Wolfgang Korte, Guichan Cao, Amy Luke, Lara R. Dugas

**Affiliations:** 1 Department of Public Health Sciences, Stritch School of Medicine, Loyola University Chicago, Maywood, IL USA; 2 Kwame Nkrumah University of Science and Technology, Kumasi, Ghana; 3 Institute of Social & Preventive Medicine, Lausanne University Hospital, Lausanne, Switzerland; 4 Ministry of Health, Victoria, Mahè Island, Seychelles; 5 Research Unit for Exercise Science and Sports Medicine, University of Cape Town, Cape Town, South Africa; 6 Solutions for Developing Countries, University of the West Indies, Mona, Kingston Jamaica; 7 Department of Nutritional Sciences, University of Wisconsin, Madison, WI USA; 8 Center for Laboratory Medicine, Canton Hospital, St. Gallen, Switzerland

**Keywords:** Hypertension, Blood pressure, Obesity, Resting energy expenditure

## Abstract

**Background:**

Obesity is a major risk factor for hypertension, however, the physiologic mechanisms linking increased adiposity to elevations in blood pressure are not well described. An increase in resting energy expenditure (REE) is an obligatory consequence of obesity. Previous survey research has demonstrated that REE is an independent predictor of blood pressure, and eliminates the co-linear association of body mass index. This observation has received little attention and there have been no attempts to provide a causal explanation.

**Methods:**

At baseline in an international comparative study on obesity, 289 participants aged 25–44 were recruited from communities in the US, the Seychelles, Ghana and South Africa and had REE measured with indirect calorimetry. All participants were thought to be free of major illness.

**Results:**

In multivariate regression models, both systolic and diastolic blood pressure were positively associated with REE (*p* < 0.01), while body mass index and fat mass were negatively correlated with systolic blood pressure (*p* < 0.01, and *p* < 0.05 respectively), but not diastolic blood pressure.

**Conclusions:**

These data confirm previous reports and suggest that a common physiologic abnormality links REE and blood pressure. Elevated catecholamines, a putative metabolic characteristic of obesity, is a possible candidate to explain this association. The direct role of excess adipose tissue is open to question.

## Background

Resting energy expenditure (REE) accounts for 70–80% of total daily energy expenditure and is tightly regulated, exhibiting relatively little variation within an individual over time. Epidemiologic survey research on the inter-relationship of REE with traits involved in the cardiovascular metabolic risk pattern and other chronic diseases has been focused primarily on the potential relationship with obesity [1]. The majority of energy required in the resting state is devoted to protein synthesis and maintenance of the electro-chemical gradient across cell membranes [2]. As a consequence, REE is highly correlated with lean body mass although it can be up-regulated by increases in circulating thyroxin and catecholamines [3–5]. REE also responds to variation in energy intake, for example, during periods of sustained fasting REE declines to compensate for lower energy intake along with profound changes in the hormone milieu [6].

Because of its important role in the energy budget, inter-individual variation in REE has been examined extensively as a potential contributor to susceptibility to obesity. A series of widely cited studies conducted among the Pima Indians in the US Southwest has reported a significant negative correlation between REE and risk of obesity [7, 8]. However, our group found equivalent REE levels after adjustment for body size and composition in a study comparing African-Americans and rural Nigerians, and no relationship to risk of future weight gain [9]. This null REE-obesity association is consistent with previously reported research finding no effect for either REE, or sleeping EE, for future weight gain among obesity-resistant or obesity-prone women [10, 11].

As a measure of metabolic activity in the resting state, it is reasonable to speculate that a range of systemic metabolic functions might be influenced by, or associated with, REE [12]. In a previous report, we identified a strong relationship between REE and blood pressure (BP) after controlling for measures of body size and composition in population-based samples in the US and Nigeria [13]. This relationship has also been described in a case-control study of obese and non-obese individuals [14], as well as in indigenous Siberians [15]. A recent report found after 4.5 years of follow-up in healthy, overweight African American and white women, that REE was a significant predictor of systolic blood pressure, independent of adiposity [12].

Adiposity has long been recognized as a risk factor for hypertension [13, 16, 17]. However, the underlying physiological mechanisms that link excess body fat stores to BP are poorly understood [13]. Obesity is a highly complex syndrome that is a consequence of metabolic alterations and lifestyle patterns that include, but are not limited to, insulin resistance, hyper-leptinemia, elevated BP, a high-calorie/high-sodium intake and reduced physical activity [13, 18]. Co-linearity of these relationships makes it difficult to isolate specific causal effects. For example, sodium intake is associated with BP in cross-sectional studies, although the effect is reduced or eliminated by control for body mass index (BMI) [19]. The increased energy intake among the obese makes higher salt consumption obligatory; whether control for BMI is “over-control” is therefore an open question [20]. Abnormalities in the renin-angiotensin system and increased levels of various regulatory hormones, like insulin and leptin, have been consistently described to take place among the obese, although the causal link to hypertension is tenuous. A number of other metabolic abnormalities, have been reported among obese vs. non-obese hypertensive individuals, although these associations are relatively inconsistent [13, 21]. Generally, it is assumed that body mass itself, driven largely by the size of body fat stores, is the primary determinant of hypertension risk. However, it must be recognized that an obligatory increase in lean body mass occurs in parallel with total body weight, which in the general population is highly correlated with fat mass [13, 21].

As part of an international comparative study of the evolution cardio-metabolic risk factors, we have been investigating the effects of energy expenditure and obesity on BP in populations of the African diaspora. Using community-based samples in Nigeria, Jamaica, and the United States, we previously demonstrated that metabolic processes represented by REE appear to mediate the effect of body size on BP [13]. In the current study, we aimed to confirm that finding in four additional independent population samples in the African diaspora. In this second phase the community-based samples were also drawn from a wide spectrum of social and cultural contexts. The findings reported here strongly confirm the association between REE and BP.

## Methods

### Participant recruitment

The Modeling the Epidemiologic Transition Study (METS) is an international comparative study investigating the associations between physical activity and diet, and weight gain in young African-origin adults (25–45 years) [22–25]. A very detailed description of the study protocol has been previously published [26]. At baseline; twenty-five hundred adults were enrolled between January 2010 and December 2011. Five hundred participants, about 50% female, were recruited from each of the five sites of interest: rural Ghana, urban South Africa, Seychelles, urban Jamaica, and metropolitan Chicago [26]. The current analysis included a sub-set of participants with valid REE and BP measurements; participants from Jamaica were not included in these analyses due to lack of valid REE measurements. Participants were excluded from the study if they had or developed any infectious diseases, including HIV-positive individuals, pregnant or lactating women, and people with conditions preventing them from participating in normal physical activities [27].

METS protocol was approved by the Institutional Review Board of Loyola University Chicago, IL, US; the Committee of Human Research Publication and Ethics of Kwame Nkrumah University of Science and Technology, Kumasi, Ghana; the Health Sciences Faculty Research Ethics Committee of the University of Cape Town, South Africa; the Board for Ethics and Clinical Research of the University of Lausanne, Switzerland; the National Research Ethics Committee of Seychelles; and the Health Sciences Institutional Review Board of the University of Wisconsin, Madison, WI, USA. Written informed consent was obtained from all participants [26].

### Measurements

Measurements were gathered at outpatient clinics located in the respective communities. BP measurements were performed by trained and certified observers, according to standard procedures, and as previously described [28]. Briefly, measurements were made in the sitting position, with the arm at heart level after a 5-min rest [26]. An oscillometric device, previously evaluated in our field settings, was used for all BP measurements (Omrom HEM-412). A total of six measurements were performed. Initially three measurements were taken 3 min apart, and a further three measurements were repeated 1 h later, also following a 5-min rest period. For the analysis, BP was calculated as the average of 4 measurements; 2, 3, 5, and 6 [26]. Weight (kg) and height (m) measurements were performed while participants wore light clothing and no shoes, and according to the same procedures across the 5 sites, including identical stadiometer’s (Invicta, Germany), and using the same model standard calibrated balance at all five sites (Seca 770, Hamburg, Germany) [26]. BMI was calculated as kg/m^2^. Body composition was approximated by bioelectrical impedance analysis (BIA) with the use of a single-frequency (50 kHz) impedance analyzer (model BIA 101Q; RJL Systems, Clinton Township, MI), using a tetrapolar placement of electrodes was used on the right hand and foot. Fat-free mass (FFM) and fat mass (FM) were estimated from measured resistance by using an equation validated in the METS cohorts [29].

#### Body composition (isotope dilution)

Total body water was measured using isotope dilution in a subset of 75 participants at baseline in each site, and as previously described [26, 30]. The basis of this measurement is the dilution principle: total body water was calculated using the measurement of the abundance of either isotope (deuterium or 18-oxygen) from a doubly labeled water (DLW) procedure after complete equilibration with body water [31] and correction for non-aqueous exchange of 1.042 and 1.007, respectively [32]. Fat-free mass was calculated using a hydration constant (0.732 [33]) from total body water, and fat was calculated as the difference between body weight and fat-free mass [34].

#### Resting energy expenditure

REE was measured in the DLW subsample using indirect calorimetry [26]. In the US, Ghana, and Seychelles sites, REE was measured using the Cosmed Quark RMR indirect calorimeter (Cosmed USA, Chicago, IL, USA); and in South Africa, the VMax indirect calorimeter by SensorMedics (Viasys Health Care, Waukegan, IL, USA) was used. Cross-validation of instruments was carried out through external calibrations. The investigators have had extensive experience in the measurement of REE across multiple sites, with over 2500 measurements made in the US and abroad [9, 35, 36].

The detailed description of measurement of REE using indirect calorimetry has been previously published [35–37]. Participants were asked to fast from 10 pm the evening prior to the initial examination and were rested for at least 15 min prior to the REE measurement. Respiratory gases were collected for 30 min, the first 10 min of data were discarded and last 20 min used to estimate REE. Oxygen and carbon dioxide were continuously sampled during the procedure and minute-by-minute consumption and production values were calculated; energy expenditure was calculated according to the modified Weir equation [38]. REE data were available for 289 participants.

#### Physical activity (accelerometer)

Physical activity (PA) was measured using the Actical accelerometer (Phillips Respironics, Ben, OR, USA), and as previously described [26]. Briefly, participants wore the monitor at the level of the waist, positioned just behind the right hip [26]. All participants were asked to wear the activity monitor at all times over an 8-day period encompassing part of the first and last days the monitor was worn, and to not take the monitor off during sleep. Using the same protocol employed by the National Center for Health Statistics for the analysis of accelerometry data in the continuous National Health and Nutrition Examination Survey [39], minutes were defined as comprising sedentary (<100 counts per minute [cpm]), moderate (1535–3959 cpm), vigorous ≥3960 cpm or a combination of moderate-plus-vigorous activity, using published cut-points [40, 41]. PA data are presented as the total time in minutes accumulated in either 1- or 10- min intervals; the 10-min interval may be considered a modified 10-min bout as we allowed for up to 2 min of below threshold count activity before considering the bout to be ended [39].

#### Biochemical measures

Participants were asked to fast from the evening prior to the baseline clinic examination, and as previously described [26]. Fasting blood samples were drawn for analysis of adipose-related hormones (leptin, adiponectin) and other analytes (glucose and insulin). Briefly, blood samples were processed and plasma or serum separated within two hours of collection and stored at −80 °C in the laboratory at each study site [26]. Fasting plasma glucose was measured using the glucose oxidase method at each site at the time of collection. Insulin, leptin and adiponectin from all sites were measured using radioimmunoassay kits at the departmental laboratory at Loyola University Chicago (Linco Research, Inc., St. Charles, MO). All remaining assays were conducted at the Zentrum fϋr Lambormedizin, Leiter Klinische Chemie und Hämatologie**,** St. Gallen, Switzerland.

#### Questionnaires

All questionnaires were administered by centrally trained personnel, and as previously described [26]. These included necessary health history information, with a focus on cardiovascular disease (CVD) conditions and diabetes. Information included age of first diagnosis where applicable, and medication and dietary supplement use.

In total, 54 additional questions were included which covered general household characteristics, participant and significant other’s occupation, parental education and household assets and amenities, and as previously described [26]. These questions were based on the Core Welfare Indicators Questionnaire from the World Bank, designed to monitor social indicators in the Human Development Index (HDI) contexts [42].

Loyola University Chicago (Chicago, IL, USA) is the centralized coordinating center for METS [26], Briefly, all data forms, including the questionnaires were scanned at each study site and along with electronic Actical data files, sent via secure FTP (Bitvise Tunnelier [43]) to the data manager at the coordinating center.

#### Statistical analysis

Participant characteristics were summarized using means ± standard deviations (SD) and proportions. For hypothesis testing of continuous variables we employed a two-way Anova with Bonferroni correction, multiple linear regression analysis, using dummy variables for site and Pearson’s Correlation Coefficient. For categorical variables, we used the Chi-Square statistic. An alpha *p*-value of 0.05 was used to denote statistical significance. The statistical analyses was performed using Stata (version 12, College Station, TX).

## Results

For the present study, the analytical data set included all participants who underwent REE and body composition measurements at the same time, and had valid BP measurements. The final sample included 289 participants of which 69 were from Ghana, 72 from South Africa and 72 from Seychelles, and 76 from the US.

Baseline participant characteristics are presented in Table 1. Women were slightly older than men (33.6 ± 5.9 vs. 35.0 ± 6.0 yrs., *p* < 0.05), and at all sites had on average higher BMI than men and in all but 1 site weighed more than men. As expected, men had higher mean fat free mass levels. Men also had higher mean systolic and diastolic BP measurements. The mean systolic BP (SBP) among men was 121.9 ± 15.4 mmHg, and among women was 111.4 ± 16.2 mmHg. The mean diastolic BP (DBP) among men was 73.3 ± 12.2 mmHg, and among women was 71.8 mmHg. Men and women from the US, and South Africa presented with the highest SBP and DBP’s. Women presented with higher resting pulse values (*p* < 0.001), but which were similar across sites among both men and women (*p* = NS). With regards to PA levels, across all sites, participants spent approximately 33.9 ± 27.7 min/d in moderate-and-vigorous activity (1-min bouts), very little time doing any vigorous activity (3.4 ± 6.8 min/d) and approximately 4 h a day being sedentary (228.5 ± 50.8 min/d).

**Table 1:** Participant Characteristics by Site: Mean ± SD

	Ghana	South Africa	Seychelles	United States
Men				
Age (y)	35.6 ± 6.1	33.0 ± 6.2	33.4 ± 5.3	32.9 ± 5.8
Weight (kg)	62.4 ± 7.1**	62.5 ± 12.6**	73.0 ± 11.2*	90.9 ± 24.2
Fat Free Mass (kg)	53.6 ± 5.9**	47.4 ± 7.1**	55.2 ± 5.6	63.7 ± 10.1
Body Fat (%)	14.4 ± 5.2	23.4 ± 6.1	23.3 ± 9.0	27.6 ± 11.8
Body Mass Index (kg/m^2)	22.0 ± 2.3**	22.1 ± 4.1**	25.2 ± 3.6*	28.5 ± 7.7
Systolic Blood Pressure (mmHg)	116.8 ± 10.5**	121.1 ± 20.6	119.4 ± 13.4	128.7 ± 14.0
Diastolic Blood Pressure (mmHg)	66.7 ± 10.1**	72.0 ± 14.1*	71.7 ± 10.7**	80.9 ± 9.7
Resting hear rate (bpm)	63.2 ± 8.0	62.0 ± 6.8	60.5 ± 8.3	61.3 ± 8.4
Resting Energy Expenditure (kcal/day)	1618 ± 136	1528 ± 202**	1651 ± 170	1768 ± 371
MVPA (min/day)	60.0 ± 23.1*	52.5 ± 25.4	49.2 ± 31.1	38.9 ± 33.1
Women				
Age (y)	37.4 ± 6	34.2 ± 6.0	32.1 ± 6.3**	34.3 ± 5.8
Weight (kg)	63.2 ± 15.9**	83.9 ± 26.4	75.8 ± 18.6*	88.9 ± 18.6
Fat Free Mass (kg)	41.3 ± 6.6**	45.6 ± 8.5	43.6 ± 6.6	49.3 ± 7.3
Body Fat (%)	32.9 ± 9.3	43.2 ± 10.3	41.0 ± 7.6	43.1 ± 10.8
Body Mass Index (kg/m^2)	25.4 ± 7.0**	33.0 ± 9.4	28.4 ± 6.2*	33.2 ± 7.4
Systolic Blood Pressure (mmHg)	105.6 ± 12.8**	116.5 ± 19.7	105.2 ± 8.5**	117.8 ± 16.7
Diastolic Blood Pressure (mmHg)	63.0 ± 11.2**	73.0 ± 12.6**	69.7 ± 7.6**	81.5 ± 13.3
Resting heart rate (bpm)	73.6 ± 8.8	72.7 ± 11.0	68.9 ± 7.5	70. ± 9.5
Resting Energy Expenditure (kcal/day)	1389 ± 167	1550 ± 232	1404 ± 190	1439 ± 294
MVPA (min/day)	26.7 ± 17.3	18.6 ± 16.1	20.4 ± 12.9	18.3 ± 21.9

*p < 0.05 compared to US, **p < 0.01 compared to the US

Biochemical results are presented in Table 2. The majority of participants presented with healthy biochemical measures. The mean glucose levels across all sites were 93.2 ± 19.1 mg/dL, with participants in Ghana presenting with the highest values (100.6 ± 9.7 mg/dL). Mean insulin levels were 17.6 ± 10.5 mlU/L and lowest among the Ghanaians (13.7 ± 6.0 mlU/L). CR*P* values were highest among the 2 African sites, compared to the US and Seychelles, whereas measures of lipid metabolism were lowest among South Africans and Ghanaians.

**Table 2:** Participant Biochemical Measures by Site, mean ± SD

	Ghana	South Africa	Seychelles	United States
Men				
Glucose (mg/dL)	102.5 ± 10.0	83.1 ± 16.1**	98.4 ± 13.9	95.8 ± 11.9
Insulin (μIU/mL)	11.7 ± 3.8*	11.2 ± 5.2*	14.5 ± 6.7	17.4 ± 12.9
HOMA-IR	2.9 ± 0.9	2.3 ± 1.3**	3.6 ± 1.9	4.3 ± 3.5
Leptin (ng/mL)	5.2 ± 5.3	5.6 ± 5.1	8.4 ± 7.4	10.8 ± 13.8
Adiponectin (ng/mL)	9677.3 ± 4042.9	9854.9 ± 4211.3	5590.3 ± 2863.7*	8544.0 ± 4651.7
CRP (mg/L)	4.3 ± 11.4	2.7 ± 3.8	1.5 ± 1.2	2.2 ± 2.3
Total cholesterol (mg/dL)	159.5 ± 40.6	171.1 ± 40.3	164.2 ± 39.4	175.2 ± 35.4
LDL cholesterol (mg/dL)	93.8 30.1	97.9 ± 32.4	89.5 ± 27.8	105.6 ± 33.3
HDL cholesterol (mg/dL)	47.3 ± 21.7	58.0 ± 25.2	54.1 ± 14.3	50.5 ± 13.5
Triglycerides (mg/dL)	90.3 ± 38.4	94.3 ± 43.9	75.7 ± 40.7	96.1 ± 58.4
Women				
Glucose (mg/dL)	98.9 ± 9.2	83.7 ± 15.5	89.4 ± 18.7	95.4 ± 34.5
Insulin (μIU/mL)	15.3 ± 7.0**	22.4 ± 13.7	19.6 ± 9.0	24.4 ± 14.3
HOMA-IR	3.7 ± 1.9*	4.8 ± 3.5	4.3 ± 2.4	5.7 ± 3.7
Leptin (ng/mL)	30.7 ± 25.6	37.0 ± 26.7	39.4 ± 24.1	41.5 ± 20.1
Adiponectin (ng/mL)	10,352.1 ± 4502.9	11,595.5 ± 6222.8	8005.7 ± 5134.3	9461.5 ± 5389.5
CRP (mg/L)	7.6 ± 20.0	12.5 ± 17.5	5.2 ± 5.3	5.5 ± 4.6
Total cholesterol (mg/dL)	164.0 ± 33.0*	157.0 ± 31.1**	169.0 ± 34.5	187.6 ± 36.7
LDL cholesterol (mg/dL)	102.3 ± 28.3	96.4 ± 30.0*	106.8 ± 38.0	119.0 ± 37.2
HDL cholesterol (mg/dL)	44.6 ± 11.7	45.1 ± 15.2	48.4 ± 8.6	49.7 ± 13.0
Triglycerides (mg/dL)	86.7 ± 39.5	78.5 ± 17.5	63.9 ± 43.2*	96.7 ± 56.5

*p < 0.05 compared to the US, **p < 0.01 compared to the US

Data concerning participants’ medical background and behaviors are presented in Table 3. Overall, 12.8% of the participant sample had hypertension. Among men; 24% of Americans were hypertensive compared to 18% for Seychellois, 11% for South Africa and only 3% among Ghanaians (*p* = 0.094). Similarly, 24% of US women were hypertensive compared to 16% of South Africans, 3% of Seychellois and Ghanaian women (*p* < 0.01). 45.7% of the participant population met the U.S. General Guidelines for PA. As expected, a significantly higher percentage of men met the U.S. General Guidelines than women (65 vs. 35%, *p* < 0.001). 31.6% of the participant sample indicated that they smoke, with more men indicating that they smoked compared to women (45.4 vs. 14.5%). Approximately 46.7% of the participant population reported that they consumed alcohol, with twice as many men indicating alcohol consumption than women (64.6 vs. 32.1%). Men on average reported consuming 1.8 ± 3.0 units/day and women reported 0.68 ± 1.7 units/day.

**Table 3:** Participant Categorical Data - *n*, %

	Ghana	South Africa	Seychelles	United States	p-value
Men					
Overweight/Obese	28 (90.32)	23 (82.14)	17 (51.52)	18 (47.37)	0.001
Hypertension	1 (3.23)	3 (10.71)	6 (18.18)	9 (23.68)	0.094
Meets PA Guidelines	27 (87.10)	22 (78.57)	23 (69.70)	21 (55.26)	0.025
Smoker	0 (0.00)	18 (58.10)	8 (24.24)	24 (63.16)	0.001
Drinker	12 (38.71)	19 (67.86)	27 (81.82)	26 (68.42)	0.003
Women					
Overweight/Obese	22 (57.89)	14 (31.82)	11 (28.21)	4 (10.53)	0.001
Hypertension	1 (2.63)	7 (15.91)	1 (2.56)	9 (23.68)	0.006
Meets PA Guidelines	17 (44.74)	8 (18.18)	14 (35.90)	9 (23.68)	0.042
Smoker	0 (0.00)	6 (13.64)	1 (2.56)	13 (34.21)	0.001
Drinker	3 (7.89)	12 (27.27)	22 (56.41)	14 (36.84)	0.001

Partial correlations were examined between REE, SBP, DBP, pulse, weight, fat-free mass, and age for men and women and are represented in Table 4. Among men, REE (kcal/d) was significantly correlated with both SBP and DBP (mmHg), weight (kg), fat free mass (kg) and the adipokines (leptin and adiponectin). Similarly, among women, REE was significantly correlated with the same variables, and additionally pulse (bpm), but not adiponectin.

**Table 4:** Partial Correlations between REE, Age, Systolic and Diastolic BP, resting heart rate (HR), Weight, Fat Free Mass, and Adipokines for Men and Women (*P* Values)

				Men						
	REE	Age	SBP	DBP	Pulse	Weight	FFM	Insulin	Leptin	Adiponectin
REE										
Age	−0.0065 (0.9415)									
SBP	**0.2666 (0.0022)**	0.1860 (0.342)								
DBP	**0.2570 (0.0032)**	**0.1950 (0.0262)**	**0.8661 (0.0000)**							
Resting HR	0.1263 (0.1521)	0.0993 (0.2609)	0.0242 (0.7843)	**0.1849 (0.0352)**						
Weight	**0.7069 (0.0000)**	0.0615 (0.4873)	**0.2596 (0.0029)**	**0.3813 (0.0000)**	0.0336 (0.7040)					
FFM	**0.6755 (0.0000)**	0.0374 (0.6731)	**0.1810 (0.0393)**	**0.2064 (0.0185)**	−0.0420 (0.6355)	**0.8431 (0.0000)**				
Insulin	**0.3479 (0.0001)**	0.0827 (0.3555)	0.1388 (0.1198)	**0.2532 (0.0041)**	0.1417 (0.1121)	**0.3674 (0.0000)**	**0.2214 (0.0123)**			
Leptin	**0.4426 (0.0000)**	0.0621 (0.4843	0.1135 (0.2001)	**0.2522 (0.0039)**	0.1236 (0.1628)	**0.7313 (0.0000)**	**0.4841 (0.0000)**	**0.3631 (0.0000)**		
Adiponectin	**−0.2115 (0.0157)**	0.1146 (0.1943)	0.0190 (0.8299)	−0.0714 (0.4194)	−0.0949 (0.2829)	**−0.2330 (0.0076)**	−0.1282 (0.1462	**−0.2857 (0.0011)**	**−0.2351 (0.00073**	
				Women						
	REE	Age	SBP	DBP	Pulse	Weight	FFM	Insulin	Leptin	Adiponectin
REE										
Age	0.0262 (0.7426)									
SBP	**0.2023 (0.0105)**	**0.2674 (0.0007)**								
DBP	**0.2475 (0.0017)**	**0.1817 (0.0219)**	**0.8451 (0.0000)**							
Resting HR	**0.2234 (0.0047)**	−0.0634 (0.4269)	−0.0413 (0.6050)	0.0435 (0.5858)						
Weight	**0.6264 (0.0000)**	0.0155 (0.8461)	**0.1298 (0.1030)**	**0.3597 (0.0000)**	0.0447 (0.5762)					
FFM	**0.6701 (0.0000)**	0.0248 (0.7567)	**0.2140 (0.0068)**	**0.3287 (0.0000)**	0.0182 (0.8202)	**0.8058 (0.0000)**				
Insulin	**0.4378 (0.0000)**	−0.0699 (0.3893)	0.0408 (0.6153)	**0.2691 (0.0007)**	**0.1947 (0.0155)**	**0.6333 (0.0000)**	**0.4727 (0.0000)**			
Leptin	**0.3230 (0.0000)**	−0.0354 (0.6625)	−0.1160 (0.1518)	0.1135 (0.1612)	0.0833 (0.3044)	**0.7069 (0.0000)**	**0.4264 (0.0000)**	**0.4841 (0.0000)**		
Adiponectin	−0.1556 (0.0539)	0.1171 (0.1482)	0.0344 (0.6717)	−0.1089 (0.1788)	−0.0150 (0.8537)	**−0.2852 (0.0003)**	**−0.2643 (0.0009)**	**−0.3284 (0.0000)**	**−0.2069 (0.0100)**	

Multiple regression analyses were conducted to determine relationships between BP and REE, age, gender, body mass index, fat free and fat mass, resting pulse, and location of site. We present two models, Model 1 included BMI, and Model 2 included fat and fat free mass levels. These results are shown in Table 5. For Model 1, after adjusting for BMI, age, gender, pulse, and site, having higher REE (kcal/d) was significantly associated with greater systolic BP (β = 0.019, *p* = 0.001) and strongly associated with diastolic BP (β = 0.006, *p* = 0.056) BP (mmHg). Similarly, adjusting for fat and fat free mass, instead of BMI, indicated that those with higher REE (kcal/d) also had significantly higher systolic BP (β = 0.019, *p* = 0.001) and a strong association with diastolic BP (β = 0.007, *p* = 0.056).

**Table 5:** Multiple Regression analysis between blood pressures, resting energy expenditure, age, gender, BMI, fat- and fat-free mass, resting heart rate (HR), physical activity (MVPA) levels and sites

	Model 1						Model 2					
	Systolic BP			Diastolic BP			Systolic BP			Diastolic BP		
	Coefficient	SE	P-value	Coefficient	SE	P-value	Coefficient	SE	P-value	Coefficient	SE	P-value
n	264			264			264			264		
Intercept	82.15	10.1	0.001	36.402	7.480	0.001	76.918	10.596	0.001	38.551	7.822	0.001
Male	4.71	2.86	0.001	3.047	2.109	0.150	3.933	3.205	0.221	3.482	2.366	0.142
Age (years)	0.797	0.153	0.001	0.554	0.113	0.001	0.782	0.153	0.001	0.564	0.113	0.001
REE (kcal/d)	0.019	0.004	0.001	0.006	0.003	0.056	0.019	0.005	0.001	0.007	0.004	0.056
BMI (kg/m^2^)	−0.443	0.164	0.008	0.102	0.121	0.400	―	―	―	―	―	―
Fat Free Mass (kg)	―	―	―	―	―	―	0.018	0.170	0.916	−0.054	0.126	0.663
Fat Mass (kg)	―	―	―	―	―	―	−0.237	0.092	0.011	0.060	0.069	0.382
Resting HR (bpm)	−0.064	0.106	0.549	0.179	0.078	0.023	−0.055	0.107	0.606	0.173	0.079	0.030
MVPA (min/d)	−0.033	0.039	0.388	−0.024	0.029	0.402	−0.035	0.039	0.365	−0.024	0.029	0.402
Ghana	−14.240	2.800	0.001	−16.167	2.064	0.001	−15.558	3.001	0.001	−16.056	2.215	0.001
South Africa	−4.440	2.480	0.075	−8.046	1.829	0.001	−4.867	2.597	0.062	−8.178	1.917	0.001
Seychelles	−10.720	2.596	0.001	−8.862	1.916	0.001	−11.474	2.678	0.001	−8.814	1.977	0.001

## Discussion

In this report, we found an independent association between REE and BP among five populations with different mean BP levels. As noted earlier, the obesity syndrome should be conceptualized as a complex, inter-correlated set of external exposures and internal physiologic responses [13, 18]. Separating cause of effect relationships is therefore inherently difficult. Our findings suggest that the measure of REE captures a physiologic process more tightly associated with BP than basic estimates of weight-for-height, as modeled by BMI. The most likely candidate linking REE with BP is circulating catecholamines [44]. Although inconsistent, a substantial body of literature has demonstrated elevated catecholamines in hypertensive subjects [45–47]. Some investigators have likewise argued that increased sympathetic activity secondary to hyperinsulinemia is a feature of the obesity syndrome [48, 49]. Palatini et al. [50] found that among 3 international adult cohorts, fasting insulin and post 75-g glucose load correlated positively with higher heart rates. There is also some evidence that obese adults are more likely to present with increased urinary noradrenaline levels [51] and plasma levels [52]. Weight loss studies provide evidence supporting the catecholamine hypothesis in obesity. Among twenty-five obese individuals studied during a short-term weight loss intervention, a reduction in plasma norepinephrine was closely associated with reductions in REE and BP [14]. Leptin has also been associated with thermogenesis and hypertension and is markedly elevated in the obese [53]. While not a significant predictor in the current study, the role of other inflammation markers such as CRP has also been cited as a significant predictor of future CVD. For example, several studies find a relationship between adipocytokines, CRP and risk for CVD [54, 55]. Seven et al. [54] explored the relationship between leptin, adiponectin, CRP and risk for CVD in over 6500 adults (30-60 yrs). After 11 years of follow-up only CRP was found to be significantly associated with an increased CVD risk. Similarly, Barbero et al. [55] in a meta-analysis of almost 105,000 adults followed for 5 years, found that CRP, together with history of diabetes, past myocardial infarction, and being male were the most significant predictors for future CVD.

In attempting to disentangle correlated physiologic relationships, it can be useful to consider contextual patterns to determine whether they support or contradict the specific hypothesis being examined. REE would require increased cardiac output, which in the long-term could predispose to hypertension [56, 57]. For example, catecholamines increase with age, as does BP, and elevated heart rate has long been recognized as a predictor of hypertension [56]. Physical activity and training effects, however, are inconsistent with a straightforward causal link between REE and BP. REE tends to increase with aerobic fitness, while BP and pulse rate decline [58, 59]. Likewise, weight lifters and other athletes have marked increases in lean body mass, but avoid the obesity-related effect of high BP [60]. We have also demonstrated that the association between BMI and BP is weaker among the obese than the non-obese, suggesting that lean body mass is the more influential component of total body size [61]. It is nonetheless possible that a catecholamine-mediated effect that leads to increases in both REE and BP, could be over-ridden by other compensatory mechanisms after aerobic training. A simpler non-mechanistic explanation of why REE remains significantly associated with BP in our analyses, and BMI is not, could be a result of differential precision of the measurements. BMI captures about 40–50% of the variation in lean body mass, while repeat REE measurements for that person have a correlation of 0.95, reflecting very high precision. In multivariate models, a secondary variable measured with little error can achieve significance “independently” of the underlying causal effect, particularly if other covariates in the model, which may have a true causal link with the outcome, lack precision.

In our study, while we attempted to ensure consistency and reliability of measurements, particularly with regards to the measurement of BP and REE across our research sites, our study is not without limitations. For example, we have no measure of circulating catecholamines, an issue which we have now addressed in our current studies. In order to maximize comparability of REE measurements we used the same indirect calorimeter in three of the four research sites, and conducted regular methanol burns as described by Cooper et al. [62]. With regards to the measurement of BP, the same model Omron was used in all sites and all clinic staff underwent the same in-house training prior to the start of the data collection period, and as previously described by Luke et al. [26]. Due to the nature of multi-site research, there is always the potential for site-to-site variability in some measures. The investigators of the current study worked hard to minimize that variability.

## Conclusion

In summary, our study provides further evidence of a strong association between REE and BP, and a null or modest role of BMI or other adiposity measures on BP when REE is considered, which has not been fully appreciated so far. These findings expand our understanding of the complex of metabolic and physiologic abnormalities observed in the obesity syndrome.

## Abbreviations

°C: Celsius
BMI: Body mass index
BP: Blood pressure
Cm: Centimeter
CPM: Counts per minute
DLW: Doubly labeled water
FFM: Fat free mass
FM: Fat mass
FTP: File transfer program
HDI: Human Development Index
HIV: Human Immuno-deficiency Virus
IL: Illinois
Kcal/d: Kilocalories per day
Kg: Kilogram
METS: Modeling the Epidemiologic Transition Study
Min/d: Minutes per day
PA: Physical activity
REE: Resting energy expenditure
US: United States
WI: Wisconsin
Β: Beta statistic
